# Jacalin-Curcumin Complex Sensitizes the Breast Cancer MDA-MB-231 Cell Line

**DOI:** 10.3390/ijms242417399

**Published:** 2023-12-12

**Authors:** Lidiya Petrova, Nikolay Gergov, Marie Stoup, Silvina Zapryanova, Els J. M. Van Damme, Nicolas Lebègue, Maxime Liberelle, Diana Zasheva, Vanya Bogoeva

**Affiliations:** 1 Department of Biology, Medical University—Pleven, “St. Kliment Ohridski” Str. 1, 5800 Pleven, Bulgaria; lidiia.petrova-trifonova@mu-pleven.bg; 2 Institute of Molecular Biology “Rumen Tzanev”, Bulgarian Academy of Sciences, “Acad. George Bonchev” Str., Bl. 21, 1113 Sofia, Bulgaria; nikolaygergov.bio@gmail.com; 3 School of Pharmacy, University Lille, Inserm, CHU Lille, UMR-S 1172–LiNC–Lille Neuroscience and Cognition, F-59000 Lille, France; marie.stoup@univ-lille.fr (M.S.); nicolas.lebegue@univ-lille.fr (N.L.); maxime.liberelle@inserm.fr (M.L.); 4 Institute of Biology and Immunology of Reproduction, Bulgarian Academy of Sciences, Tsarigradsko Shosse, 73, 1113 Sofia, Bulgaria; silvina_z@abv.bg (S.Z.); zasheva.diana@yahoo.com (D.Z.); 5 Department Biotechnology, Ghent University, Proeftuinstraat 86, 9000 Gent, Belgium; elsjm.vandamme@ugent.be

**Keywords:** protein–ligand interaction, curcumin, jacalin, fluorescence, cytotoxicity, breast cancer cells, MDA-MB-231

## Abstract

Protein–drug interactions are crucial for understanding drug delivery and cell functions. Jacalin is a suitable molecule for such targeting, as it specifically recognizes the tumor-associated Thomsen–Friedenreich (TF) antigen that is expressed on the glycosylated proteins in cancer cells. The present paper describes the interaction of curcumin and jacalin, a possible carrier molecule for the delivery of antitumor drugs due to its ability to recognize tumor cells. Our results have shown that both steady-state fluorescence and fluorescent labelling of jacalin are two reliable methods to determine jacalin-curcumin interactions. The affinity of jacalin for curcumin is consistently within the micromolar range (using fluorescence and microscale thermophoresis) showing high-affinity binding of the complex. In vitro experiments on triple-negative breast cancer MDA-MB-231 cells indicated inhibition of cell growth after treating with the jacalin-curcumin complex for 48 h. The cell survival fraction was significantly reduced to 50% after combined treatment. In this paper, we report for the first time about the jacalin-curcumin interaction. We quantified this unique biomolecular interaction and gathered additional information on the binding event. We observed that the jacalin-curcumin complex inhibits the proliferation of the triple-negative breast cancer MDA-MB-231 cells.

## 1. Introduction

Cancer is still one of the primary diseases that cause death in the world. According to the World Health Organization, it is the second cause of death, leading to an estimated 10 million deaths globally in the last years. Breast cancer is one of the five known cancers among women. Treatment includes surgical intervention, radiation, and chemotherapeutic drugs, which destroy not only cancer cells but also healthy ones. Therefore, researchers are looking for new alternatives, including natural compounds, some of which have shown proven anticancer activity.

Recently, natural therapy has become very promising as a curative treatment, for which it is attracting scientific attention [1] compared to “adjuvant therapy” for breast cancer, which causes severe side effects in the patients and is costly. Fruits and vegetables are a source of various phytochemicals that have a chemo-preventive ability at several steps of carcinogenesis [2,3].

Jacalin is isolated from *Artocarpus integrifolia* (jackfruit, used as a fruit in Asia) [4]. It is a tetrameric protein with a molecular weight of 66 kDa that has various biological activities. The lectin, extracted from jackfruit (*Artocarpus heterophyllus*) [5], specifically targets the Thomsen–Friedenreich antigen (TF, -Gal*β*1-3GalNAc) [6] and is non-cytotoxic for normal cells. This TF antigen is expressed in more than 85% of human carcinomas. Expression of CD176 (TF antigen) is established in the MDA-MB-231 cell line (triple-negative breast cancer cells) in 5–30% of the cells [7]. Due to its TF specificity, jacalin is considered a promising drug-delivery molecule to target cancer cells [8,9,10,11,12,13].

Recently, the complex of jacalin-PEG phthalocyanine gold nanoparticles was investigated. The observed high phototoxicity reported for HT-29 cancer cells (95–98%) was explained by the specific binding of the lectin to the TF antigen, expressed onto the tumor cell membrane [8].

Curcumin is an active substance of turmeric that inhibits cell proliferation and survival. It induces apoptotic and non-apoptotic cell death and reduces invasion and migration in different types of cancer cells [14,15,16,17,18,19,20]. In a recent study, it was found that curcumin induces apoptosis in MCF-7 cells in a dose-dependent manner and that the cell survival rate depended on the curcumin concentration [21].

All these data motivated us to study the jacalin-curcumin interaction about the potential application of lectin as a drug carrier to cancer cells. Drug–protein complexes are important for biological systems [22,23,24,25,26,27,28]. Studying the molecular basis of the curcumin–protein interactions is important in designing novel therapeutic systems or improving selective drug delivery. Furthermore, we also studied the effect of the jacalin-curcumin complex on breast cancer cells. This paper is the first report about the novel jacalin-curcumin binding. We have quantified this unique biomolecular interaction, gathering additional information on the binding event. The cytotoxic effect of the complex for MDA-MB-231 cells was also investigated.

## 2. Results

**Figure 1:**
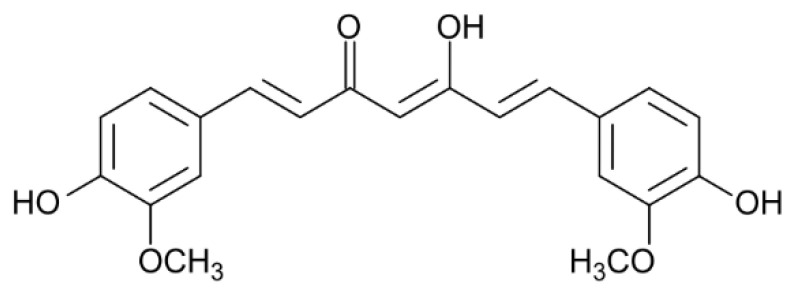
Chemical structure of curcumin.

It is reported that various plant lectins possess hydrophobic regions where they can accommodate different compounds, drugs, and physiological ligands. It has been shown that some plant lectins have multiple binding sites allowing the interaction with carbohydrate structures but also the binding with noncarbohydrate ligands [29]. Of special interest to us was to study the interaction between jacalin and curcumin (Figure 1) using steady-state fluorescence and microscale thermophoresis (MST). The present results provide novel spectroscopic and microscale thermophoresis data about the binding of jacalin with curcumin molecules.

### 2.1. Interaction of Jacalin with Curcumin, Registered by Steady-State Fluorescence

We studied the interaction between curcumin and jacalin using a fluorescence titration method. For this purpose, we performed titration of curcumin with increasing concentrations of jacalin (0.28–2.42 μM) and calculated the apparent dissociation constant K_D_ of 1.1 ± 0.22 μM, suggesting high-affinity binding of jacalin to curcumin, similar to that of jacalin–porphyrin complexes [29,30].

**Figure 2:**
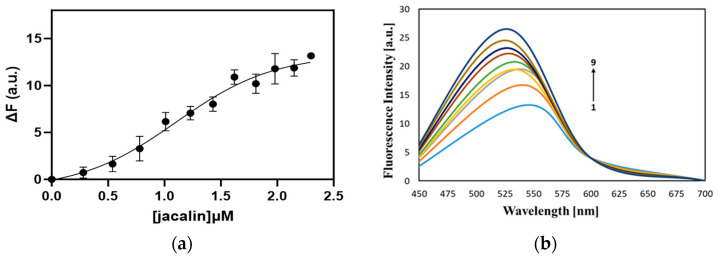
(**a**) Fluorescence titration curve, showing the interaction of jacalin with curcumin. The curve is representative of 3 experimental sets. (**b**) Fluorescence emission spectra of 1—curcumin (without jacalin); 2–9, curcumin with increasing concentrations of jacalin (0.28 μM–2.42 μM).

The fluorescence titration curve (Figure 2a) indicated the binding of nonfluorescent protein to the ligand. In Figure 2b, the emission spectra of curcumin without jacalin and with the protein are presented. The binding of jacalin to curcumin caused a significant increase in the fluorescence intensity (Figure 2b). In addition, a shift of 18 nm is observed, which is indicative of the accommodation of curcumin within the jacalin molecule.

### 2.2. Interaction of Curcumin with Fluorescently Labeled Jacalin

**Figure 3:**
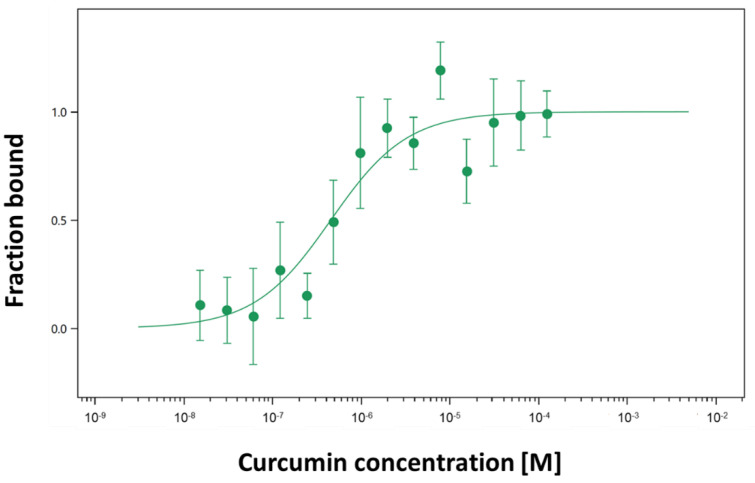
Binding of jacalin with curcumin by microscale thermophoresis (MST).

MST is a biophysical method useful to quantify the binding affinities of protein–ligand interactions, respectively, jacalin-curcumin interactions. The method uses the ability of jacalin labelled with a fluorescent tag to move along a thermal gradient. Figure 3 shows the differences in thermophoresis as a function of the curcumin concentrations and allows measuring the interaction between curcumin and jacalin. The obtained results have shown that the target protein jacalin interacts with the ligand curcumin with a K_D_ of 0.45 μM ± 0.2 (Figure 3) in the same range as for steady-state fluorescence.

### 2.3. Cell Viability Results

**Figure 4:**
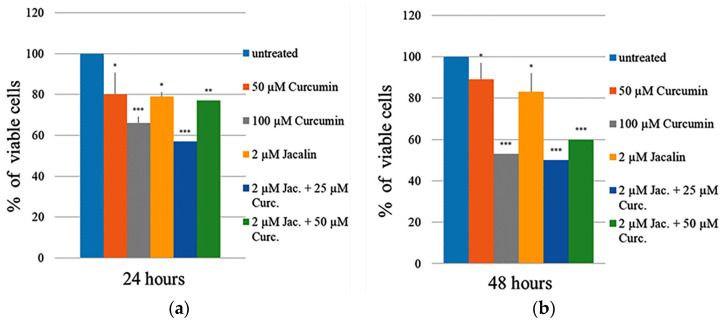
Cell viability of the MDA-MB-231 breast cancer cell line, (**a**) 24 h or (**b**) 48 h after treatment with 2 μM jacalin, 50 μM and 100 μM curcumin, and combinations of them (2 μM jacalin/25 μM curcumin; 2 μM jacalin/50 μM curcumin). Untreated samples are used as controls. The standard deviations in percentage are presented on the histogram. *—the means are statistically significant at *p* ≤ 0.05; **—the means are statistically significant at *p* ≤ 0.01; ***—the means are statistically significant at *p* ≤ 0.001.

The cytotoxic effect of jacalin (alone) and curcumin (alone), and the jacalin-curcumin complexes (with different combinations: 2 μM:25 μM, 2 μM:50 μM) were studied on the MDA-MB-231 (triple-negative breast cancer) cell line, after incubation for 24 h and 48 h (Figure 4). First, cell viability was determined after treatment with jacalin (2 μM). As shown in Figure 4a, comparatively weak cytotoxicity with a survival of about 79–83% was induced by jacalin after 24 h and 48 h incubation.

Figure 4 clearly shows the cytotoxic effect using two different concentrations of curcumin (50 μM; 100 μM). The results indicate that curcumin decreases cell growth of MDA-MB-231 cells. The experimental data show that 24 h incubation with 2 μM jacalin or 50 μM curcumin induces the same percentage (about 20%) of cell toxicity (Figure 4a). Although the curcumin concentration is 25 orders of magnitude higher than the lectin concentration, both compounds show similar effects upon the MDA-MB-231 cell line, after treating for 24 h or 48 h. Finally, the effects of jacalin-curcumin complexes (with different combinations: 2 μM:25 μM, 2 μM:50 μM) on the viability of MDA-MB-231 cells were also investigated with the MTT assay. Interestingly, the highest cytotoxicity with cell viability of 50% ± 1 was induced by the jacalin-curcumin complexes 2 μM:25 μM (Figure 4b). The treatment combination of 2μM jacalin and 50 μM curcumin is less effective, reaching a decrease of the cell survival of about 40% ± 2.

Our results confirmed the ability of the jacalin-curcumin complex to decrease the cell growth of the MDA-MB-231 line. The obtained data have shown that relatively high doses of curcumin cause a marked inhibition of the cell viability.

## 3. Discussion

A universal feature of cancer cells is altered glycosylation, and particular glycan structures are considered as glycomarkers of tumor progression. It is established that the malignant transformation is associated with significant alterations in N- and O-glycosylation processes in normal cells. In tumor cells, the aberrant O-glycans, expressed at the cancer cell surface, occur as carbohydrate components of membrane-bound N-acetyl galactosamine (O-GalNAc), glycoproteins (T and Tn antigen) and glycolipids (Sialyl-Lewis A and Sialyl-Lewis X). The aberrant O-glycans are considered as potential glycomarkers of cancer diseases [31,32]. It is reported that some lectins from plants can recognize the O-glycans on the cell surface, which makes them suitable as tools for cancer diagnosis, prognosis and therapy [33].

The plant lectin jacalin specifically targets the tumor-associated Thomsen–Friedenreich antigen and inhibits the cell viability of human colon cancer cells, displaying its potential anticancer activity [6,8,10].

Our results have shown the formation of jacalin-curcumin complexes using fluorescence spectroscopy and MST. Binding of jacalin to curcumin enhanced the emission intensity, because of the interaction (Figure 2b). The titration curve indicates the interaction of jacalin with curcumin molecule as a function of the protein concentration (Figure 2a). In our investigations, the affinity of jacalin for curcumin is in the micromolar region (K_D_ = 1.1 μM, registered by steady-state fluorescence, Figure 2a,b; K_D_ = 0.45 μM, using MST experiments, Figure 3) and it reveals that the binding of the lectin for the compound is several orders of magnitude higher than that of jacalin for porphyrins and carbohydrates [29,30]. Interestingly, the binding involves hydrophobic interactions [30,34], also for jacalin–curcumin interactions in the present study.

The carbohydrate-binding properties have been recognized as an important activity of plant lectins for many years. In addition, several plant lectins also displayed specific binding with hydrophobic ligands. These interactions occur at binding sites distinct from the carbohydrate-binding ones [25].

Jacalin binds AgNPs [12], and the interaction is similar to that of jacalin and other lectins with hydrophobic ligands and plant growth hormones, such as porphyrins, 2,6-toluidinylnapthalene-sulfonic acid, adenine, auxin and cytokinins (Ka ≈ 1.0 × 10^3^ to 1.0 × 10^6^ M^−1^) [29,30,35,36].

Jacalin is a D-galactose-specific binding lectin with a subunit molecular mass of ~16.5 kDa, composed of 133 amino acids. The X-ray-crystallographic structure of a jacalin–α-methyl-mannose complex has also been determined. Structural analyses demonstrated the presence of a large carbohydrate-binding site within the jacalin β-prism structure, which is a sufficiently flexible structural scaffold that can accommodate carbohydrate structures [37].

The protein, being a tumor-specific lectin, can bind T/Tn antigens at these carbohydrate-binding sites [38].

The jacalin structure demonstrated that native jacalin consists of the tight association of four identical protomers and that the protein can be considered as a tetravalent lectin [37]. Interaction of the protein with curcumin could also be explained with the flexible protein structure that can accommodate curcumin molecule.

Curcumin is a fluorescent compound, and its emission spectrum is affected by its microenvironment. We registered changes in the emission position (registered at 525 nm), (Figure 2b), indicating microenvironmental changes due to the lectin-binding, resulting in blue shifting (18 nm) and an increase of the specific curcumin fluorescence. Similar to our results, the emission maximum of curcumin in a hydrophobically modified starch (HMS) solution is shifted to 531 nm after encapsulating in the HMS solution. Interestingly, the anticancer activity of HMS-encapsulated curcumin is higher than that of the DMSO dissolved compound, demonstrating that the encapsulation enhances the curcumin delivery effect on cancer cells [39].

Curcumin was shown to possess an inhibitory effect on cell proliferation and induces apoptosis in several types of cancer cells [21]. Cell cytotoxicity of curcumin is different in various cancer cell types. Curcumin causes apoptosis in MCF-7 cells in a dose-dependent manner, and its anti-cancer effect on MCF-7 cells under 3D culture conditions could increase the effectiveness of the treatment. The cell survival depends on the compound concentration [21].

In our study, we evaluated the curcumin inhibition on breast cancer cells. As jacalin selectively recognizes T-antigens Galb-1–3-GalNAc [6], it can be utilized for targeted cancer therapy. This motivated us to study the effect of the jacalin-curcumin complex upon a breast cancer cell line. We chose the MDA-MB-231 cells, an invasive and poorly differentiated triple-negative breast cancer cell line which lacks estrogen receptors (ER), progesterone receptors (PR), and the human epidermal growth factor receptors (HER2). MDA-MB-231 does not respond to anticancer compounds which target these receptors [40,41,42,43], and the hormonal replacement therapy is also ineffective. Hence, MDA-MB-231 is a good model system for the evaluation of potential new therapeutic drugs and complexes.

Natural compounds are good alternatives to established anticancer drugs, and we chose to study their activity. The effects of two natural compounds of plant origin have been evaluated in our work.

First, we studied the cytotoxic effect of jacalin on cancer cells.

In a recent study, researchers found that jacalin was able to induce macrophage-mediated proinflammatory cytokine antitumor activity. Also, it is reported that cytokines were released via the NF-κB signaling pathway. This led to apoptosis in the human colon HT-29 and breast MCF-7 cell lines (MCF-7), respectively [13].

In mouse and rat bladder cancer models, jacalin demonstrated high discrimination between normal urothelium and neoplastic urothelium [44]. Recent reports announced that this lectin induced cell cycle arrest in MCF-7, in comparison to crude extract, purified jacalin and jacalin standard. IC_50_ for MCF-7 was achieved at concentration of 125 μg/mL [45].

However, in the present study, jacalin (alone) was found to inhibit slightly the cell growth of MDA-MB-231 cells. In the literature, the effect of jacalin (alone) was reversible, as the cells recovered after removing the lectin. Similar to this finding and contrary to our expectations, the cytotoxic effect of jacalin (2 μM) alone was lower after 48 h (reaching 17%), which is different from the higher effect of jacalin after 24 h (reaching 21%). Then, we evaluated the effect of curcumin treatment on the MDA-MB-231 cell line and registered a decrease of cell viability in a dose-dependent manner (Figure 4). These results are in accordance with the dose-dependent effects of curcumin on the MCF7 cell line [21].

Finally, our study showed that a combination treatment of a low jacalin concentration (2 μM) and a concentration of curcumin 13 orders of magnitude higher (25 μM) significantly reduces cell viability. Treating with the unique jacalin-curcumin complex is very promising, as both compounds are non-toxic for healthy cells, in contrast to chemotherapeutics [46]. Usually, standard chemotherapeutic drugs are effective at high concentrations for triple-negative breast cancers. Various combinations, including an anticancer drug and a natural compound, are the subject of studies on triple-negative cancer cell lines. They are applied to minimize the acquired and multi-drug resistance [47]. For example, the treatment combination of 11.65 μM curcumin and 93.95 μM melphalan reduced cell viability to 27% after 24 h and to 72.5% after 48 h in MDA-MB-231 cells [48]. Although there are serious side effects from melphalan treatment (such as iris and chorioretinal atrophy, retinal detachment, etc.), it is still applied for retinoblastoma treatment, and in 2021, it received its first approval for the treatment of adults in the USA [49].

The combined treatment of jacalin (40 μg/mL) with the established anti-cancer drug taxol (50 μM) reduced cell growth of the triple-negative MDA-MB-468 cell line to 50% after 48 h. To reduce the high-dose treatment and potential side effects, taxol is combined with the natural compound jacalin [50]. Similar to this, our study shows that jacalin-curcumin treatment causes a significant inhibition of cell growth of 50% after 48 h, using a two-times-lower concentration of curcumin (25 μM) than taxol (50 μM).

The obtained results showed synergistic effects of both compounds on the MDA-MB-231 cells. Our results confirmed the ability of the curcumin-jacalin combination to decrease the growth of the MBA-MD-231 cells, similar to the effect in MCF-7 cells [21].

According to Liu et al., the anticancer activity of curcumin in mediating the breast cancer cell proliferative rate and invasion is through down-regulating the NF-kB-inducing genes [51].

## 4. Materials and Methods

### 4.1. Materials

All reagents for cell cultures treatments and cell culture assay tests were purchased from Sigma-Aldrich (St. Louis, MO, USA). Curcumin was purchased from Sigma Aldrich. The protein jacalin was dissolved in phosphate-buffered saline (PBS) (20 mM phosphate buffer containing 0.15 M NaCl, pH 7.4) and the concentration of the protein was determined spectrophotometrically using the extinction coefficient. The concentration of curcumin was calculated by the absorbance at 422 nm (e_422_ = 3.7 × 10^5^ M^−1^ cm^−1^). Dulbecco’s Modified Eagle’s Medium (DMEM) medium, L-glutamine, fetal bovine serum (FBS), sodium pyruvate, nonessential amino acids, trypsin and PBS were obtained from Sigma-Aldrich (St. Louis, MO, USA). The antibiotic/antimycotic solution was purchased from Merck Sigma Aldrich|Sigma Life Science Absolute ethanol was purchased from Riedel-de Haën|Honeywell, Seelze, Germany. and the MTT solution (3-(4,5 dimethylthiazol-2-yl)-2, 5-diphenyltetrazoliumbromide) was obtained from Sigma-Aldrich (Darmstadt, Germany). Other chemicals were of the highest quality commercially available.

Measurements were performed on a Monolith NT.115. Proteins were covalently labeled with Red-NHS 2nd Generation labeling kit (Alexa Fluor 647), columns and normal/premium capillaries were purchased from NanoTemper GmbH (Munich, Germany).

### 4.2. Cell Culture

The breast cancer cell line MDA-MB-231 (ATCC cell culture collection N N HTB-26™) was used in the experiments. The cell line was grown at 37 °C in DMEM, high-glucose, with 4500 mg/L glucose, L-glutamine, sodium pyruvate, and sodium bicarbonate (Sigma, D6429-500 mL; Lot# RNBK4362). The DMEM medium was supplemented with a MEM Amino Acid (50x) solution without L-glutamine (Sigma, M5550-100 mL; Lot# RNBJ6705) and with a 10% FBS and antibiotic/antimycotic solution. An amino acid mix solution (from Sigma-Aldrich) was added to the medium. The cells were cultivated to 80% confluence in a CO_2_ incubator at 37 °C in an atmosphere of 5% CO_2_—95% air, and subcultured twice a week. Cells were trypsinized with a trypsin/EDTA solution. The cells were centrifuged, suspended in FBS with 5% DMSO.

### 4.3. Purification of Jacalin

Jacalin was isolated from jackfruit seeds by affinity chromatography on immobilized galactose. Briefly, dry seeds (20 g) were soaked overnight in distilled water at 2 °C and homogenized in a Waring blender in 200 mL of 0.1 M Tris/HCl (pH 7.5) containing 0.2 M NaCl. The homogenate was centrifuged at 9000 *g* for 15 min and the resulting supernatant filtered through filter paper (Whatman 3 MM). The cleared extract was applied to a column (2.5 cm × 10 cm; approx. 50 mL bed volume) of galactose–Sepharose 4B equilibrated with 0.1 M PBS (pH 7.5). After loading the extract, the column was washed with the same buffer until the A_280_ value fell below 0.01. The bound jacalin was eluted with a solution of 0.1 M galactose in PBS and dialyzed against PBS. To remove any possible trace of KM+, the lectin preparation was rechromatographed on galactose–Sepharose 4B, re-eluted with 0.1 M galactose in PBS, dialyzed against the appropriate buffer and stored at −20 °C until use. Lectin purity was investigated by SDS-PAGE using 15% acrylamide gels. The carbohydrate-binding activity of jacalin was confirmed by agglutination assays as well as hapten inhibition assays using a 2% suspension of rabbit red blood cells, as previously described [37].

### 4.4. Steady-State Fluorescence

Fluorescence spectroscopy (FS) was performed with a Shimadzu spectrofluorometer RF-5000U (Tokyo, Japan). The emission spectra of the curcumin were recorded in a 1-cm quartz cell (Hellma Analytics, Germany). The curcumin sample was excited at 445 nm with an excitation band pass of 10 nm and an emission band pass of 10 nm. Total fluorescence was calculated after correction for dilution. Absorbance was measured using a spectrophotometer (Beckman, Brea, CA, USA). All measurements were performed at 25 °C.

Curcumin–protein interactions were studied using a fluorescence titration method, ideal due to its intrinsic sensitivity and simplicity. For this aim, curcumin was titrated with increasing concentrations (0.28–2.42 μM) of jacalin. The binding of the protein to curcumin was estimated by a variation of the fluorescence emission of curcumin (excitation at 440 nm). Experimental data (from three independent experiments) were processed by a non-linear regression analysis computed with the GraphPad Prism4 program. Data were statistically analyzed by a binding model. The maximal increase of the curcumin fluorescence due to the saturation of the binding sites by the ligand (Fmax) was estimated from the titration data.

### 4.5. Jacalin-Curcumin Interaction Studies Using Microscale Thermophoresis

MST experiments were performed by the Monolith NT.115 (NanoTemper GmbH, Munich, Germany) instrument to characterize the protein–ligand interaction. The instrument is equipped with a blue/red filter set, and only the “red” channel was used. An infrared (IR) laser is coupled into the light path of fluorescence excitation and emission, and red excitation (605–645 nm, emission 680–685 nm) was used. Experiments were carried out in MST Buffer. The protein jacalin contained lysine residues. The monolith protein labelling kit Red-NHS 2nd Generation (Alexa Fluor 647) was used for fluorescent labeling of the protein jacalin following the manufacturer’s recommendations. After elution from the column, the level of the labeled jacalin was checked spectrophotometrically. After that, the titration series with non-labeled ligand curcumin was performed. MST measures equilibrium-binding events. The 16 ligand concentrations were prepared with a serial dilution and mixed with an equal volume of the fluorescent-labeled jacalin, whose concentration was fixed. At this point, all buffers were supplemented to 5% (*w*/*v*) DMSO. The final concentration of the protein was 10 nM. The mixture with increasing concentrations of curcumin and the complex was loaded into coated capillaries (NanoTemper). A typical MST experiment featured LED illumination at 100% power and IR illumination between 20% to 60% power, at 24 °C. The instrument was set following the NanoTemper instructions. All data were analyzed using M.O. Affinity Analysis version 2.3, NanoTemper.

### 4.6. Mitochondrial Dehydrogenase Assay (MTT Assay)

Cells were seeded at 10^4^ cells per well to confluence of 80% into the DMEM medium. After 24 h, the cells were treated with jacalin or curcumin and the combination of both compounds. The following concentrations were applied: curcumin (50 μM and 100 μM); jacalin (2 μM); and two combinations: jacalin 2 μM/curcumin 50 μM, and jacalin 2 μM/curcumin 100 μM. The viability was determined at two time points: 24 h, 48 h.

The MTT test followed the standard procedure (Ref. [1]). 20 μL of MTT stock solution (5 mg/mL) was added to the well and the plates were incubated for 4 h in a CO_2_ incubator. The formazan crystals were solubilized in 150 μL dimethyl sulfoxide. The color intensity was measured spectrophotometrically, using the wavelength of 600 nm (by ELISA Reader Fluostar Optima/BMG Labtech, Ortenberg, Germany). The obtained data were processed and compared to the untreated cells. The untreated samples were used as a control with 100% viability. Assays were performed at least in triplicate. The standard deviation bars were presented in percentage in the graph.

### 4.7. Statistical Analysis

All experiments of cell survival (MTT assay) were run at least in triplicate or several repeats. Data were presented as a mean value (+SD or ±SD) from three independent experiments for the MTT assay.

Statistical analyses were performed with GraphPad Prism4 software (GraphPad Software Inc., La Jolla, CA, USA). Data are presented as the mean ± SEM. Differences in the means of samples were analyzed by one-way ANOVA with selected comparisons using Tukey’s test or by two-way ANOVA with selected comparisons using the Bonferroni post hoc test, and the differences were considered significant at *p* < 0.05 *, *p* < 0.01 **, or *p* < 0.001 ***. The results are presented with standard deviation bars. The absolute values of the MTT assay of breast cancer cell line data for different treatment conditions are processed in Student’s *t*-test for two different conditions (control versus experiment).

## 5. Conclusions

In conclusion, our results have demonstrated that both steady-state fluorescence and fluorescent labelling of jacalin are two reliable methods to determine jacalin-curcumin interactions. The affinity of jacalin for curcumin is consistently within the micromolar range (using fluorescence and microscale thermophoresis), showing high-affinity binding of the complex.

Jacalin potentiates and facilitates the cytotoxic effect of curcumin. The obtained results have shown a synergistic effect of the jacalin-curcumin complex on the MDA-MB-231 cell line.

The present study characterizes the capacity of jacalin to bind curcumin; in particular, the complex could be an appropriate alternative to classical anti-cancer drugs and reveals new perspectives in the drug delivery of anti-cancer agents. The jacalin-curcumin complex could be used also for cancer prevention or as an additive to relieve the side effects of standard chemotherapeutic drugs. The analysis of the protein binding to curcumin not only displays fundamental knowledge about the biomolecular binding but may also direct the way towards improved disease treatment. This study reveals new perspectives for biomedical application of jacalin-curcumin complexes.

## Data Availability

Data is contained within the article.
